# Retrospective file review shows limited genetic services fail most patients – an argument for the implementation of exome sequencing as a first-tier test in resource-constrained settings

**DOI:** 10.1186/s13023-023-02642-4

**Published:** 2023-04-12

**Authors:** Emma K. Wiener, James Buchanan, Amanda Krause, Zané Lombard, for the DDD-Africa Study, as members of the H3Africa Consortium

**Affiliations:** 1 Division of Human Genetics, National Health Laboratory Service and School of Pathology, Faculty of Health Sciences, University of the Witwatersrand, Johannesburg, South Africa; 2 Health Economics Research Centre, Nuffield Department of Population Health, University of Oxford, Oxford, UK; 3 NIHR Oxford Biomedical Research Centre, Oxford, UK

**Keywords:** Low- and middle-income countries, Genetic services, Exome sequencing, Developmental disorders, Diagnostic guidelines

## Abstract

**Background:**

Exome sequencing is recommended as a first-line investigation for patients with a developmental delay or intellectual disability. This approach has not been implemented in most resource-constraint settings, including Africa, due to the high cost of implementation. Instead, patients have limited access to services and testing options. Here, we evaluate the effectiveness of a limited genetic testing strategy and contrast the findings to a conceivable outcome if exome sequencing were available instead.

**Results:**

A retrospective audit of 934 patient files presenting to a medical genetics clinic in South Africa showed that 83% of patients presented with developmental delay as a clinical feature. Patients could be divided into three groups, representing distinct diagnostic pathways. Patient Group A (18%; mean test cost $131) were confirmed with aneuploidies, following a simple, inexpensive test. Patient Group B (25%; mean test cost $140) presented with clinically recognizable conditions but only 39% received a genetic diagnostic confirmation due to limited testing options. Patient Group C – the largest group (57%; mean test cost $337) – presented with heterogenous conditions and DD, and 92% remained undiagnosed after limited available testing was performed.

**Conclusions:**

Patients with DD are the largest group of patients seen in medical genetics clinics in South Africa. When clinical features are not distinct, limited testing options drastically restricts diagnostic yield. A cost- and time analysis shows most patients would benefit from first-line exome sequencing, reducing their individual diagnostic odysseys.

**Supplementary Information:**

The online version contains supplementary material available at 10.1186/s13023-023-02642-4.

## Background

Genetic services focus on diagnosing genetic disorders, with the aim of individualizing management and refining risk assessment. Developmental disorders (DD) – which include neurodevelopmental disorders (NDD) and congenital anomalies, frequently form part of the clinical presentation of these disorders. The phenotypic features and genetic aetiology of DD are highly heterogeneous, and, in many cases, non-specific, making this group of disorders challenging to diagnose [1, 2]. For this reason, guidelines have been developed over time to attempt to improve the rate of diagnosis. Despite these guidelines, patients often face extended periods of uncertainty and ongoing clinical and genetic testing, with low returns, termed the diagnostic odyssey. This has significant cost and medical implications as well as profound psychological effects for patients and families [3].

In the past 10 years the introduction of exome sequencing as a routine testing strategy has changed the genetics services landscape rapidly, with significantly increased diagnostic yields [4], proven cost effectiveness [5] and improved turnaround time [6] before diagnosis. To this end, the American College of Medical Genetics (ACMG) recently released a new guideline for the diagnosis of children with congenital anomalies, global developmental delay and intellectual disability, recommending exome or genome sequencing as the first-line investigation [7]. An accurate molecular diagnosis means clinicians can understand the condition better, and therefore provide more accurate condition-guided management and surveillance, precision therapy where available [8], as well as recurrence risk assessment.

Due to these advances in genomic technology, genetic services and early or first-line exome sequencing are now well-established in most high-income countries’ health-care delivery systems. However, in line with the global trend of missing diversity in genomics research, genetic services remain a scarce and under-resourced commodity in low- and middle-income countries (LMIC), and especially on the African continent. Although genetic disorders featuring DD occur in these countries [9, 10], little has been published about patients with DD in Africa; in terms of diagnostic outcomes, genetic aetiologies, or the diagnostic processes currently in use [11]. Similarly, limited studies have focused on cost effectiveness and improved services in resource constraint settings linked to new technologies such as exome sequencing [12–14].

In this paper we present a retrospective file audit of a cohort of patients who presented to clinics of one of the largest medical genetics services in sub-Saharan Africa. The aim of this study is to characterize the patient cohort, and to assess the impact limited genetic testing has on diagnostic yield and patients’ diagnostic odysseys. These findings are contrasted to a conceivable outcome if exome sequencing were available instead.

## Results

### Characterization of the cohort

**Fig. 1:**
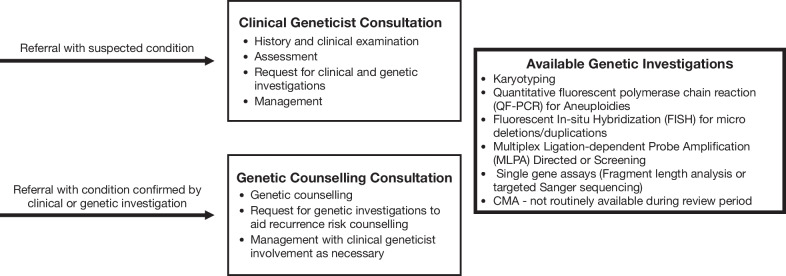
Overview of functional structure of the genetics clinic and the two main types of patient referrals

Files were retrieved for 88% (934/1059) of patients who attended a genetics clinic in 2017, offered by the Division of Human Genetics, National Health Laboratory Services (NHLS) and The University of the Witwatersrand, in Johannesburg, South Africa. These clinics operate in the University of Witwatersrand academic hospitals that are part of the public health sector, which serves 80% of the South African population. Clinic consultation are with either a medical geneticist or genetic counsellor. Patients were referred for genetic testing when an applicable test was available through the Division’s diagnostic service laboratory. The available tests are summarized in Fig. 1 and included classic cytogenetic- and targeted DNA based tests. At the time of this audit, chromosomal microarray and next generation sequencing based tests were not routinely offered.

The cohort of 934 unselected patients had a male to female ratio of 1.2:1. Most patients (90%) are Black South Africans (Additional file 2: Table 1), with all 11 official language groups represented. These statistics are indicative of a South African urban population, and match census data for Johannesburg [15]. Most patients (888/934; 95%) were younger than 18 years of age, with 43% (403) presenting to the clinic before the age of one year, and 75% (703) before the age of five years.

Seven hundred and forty-two (83%) patients presented with features of DD, with the three most prevalent features being global developmental delay (36%), congenital anomalies (33%) and dysmorphic features (26%). In total, 72%, 69% and 75% respectively of patients with these individual phenotypes remained undiagnosed. Thirty-two patients (3%) were deemed to have no genetic condition or presented with a phenotype within the range of normal variation, so were excluded from further analyses. With these patients removed, the cohort was 902 patients.

**Fig. 2:**
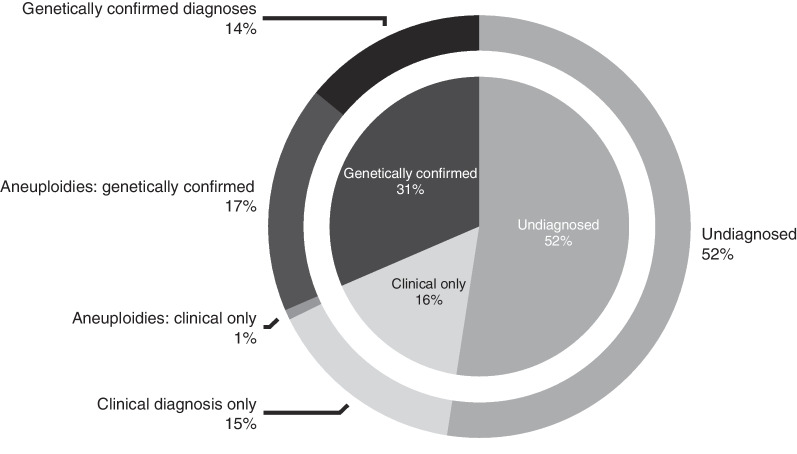
Overall percentages of patient diagnostic outcomes**.** The majority of patients remained undiagnosed, and of those with genetically confirmed diagnoses the majority were diagnosed with aneuploidies. A diagnosis was considered genetically confirmed only if a positive genetic (cytogenetic or DNA based testing methodologies) result was on record for the patient

Figure 2 shows the overall diagnostic yield achieved for this patient cohort. Half (473; 52%) of the cohort remained undiagnosed. Of the remaining patients, 16% (145) were patients who received a clinical diagnosis but no genetic confirmation of their diagnosis. Of the 31% (284) of patients who received a genetic diagnosis, a diagnosis of an aneuploidy accounted for more than half of this number (18%; 164).

### Diagnoses

There were 130 different diagnoses made, 75 of which occurred only once in this cohort. Aneuploidies accounted for four of the top 10 diagnoses (Additional file 1: Fig. 1), with Trisomy 21 being the most common, accounting for 30% (129) of all diagnoses.

**Fig. 3:**
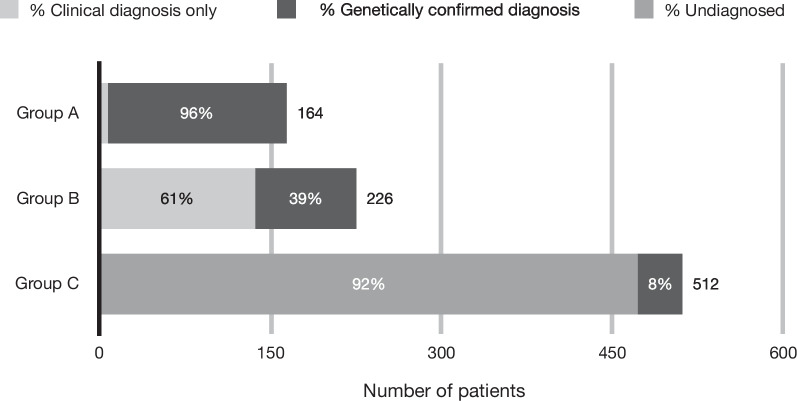
Three main groups of patients from the file review identified with differing diagnostic profiles**.** Group A: Patients with aneuploidies, Group B: Patients with easily recognizable conditions, Group C: Patients with rare, less-recognizable conditions

When considering the types of diagnoses made in this cohort three distinct groups of patients emerged, based on their different diagnostic profiles (Fig. 3),

### Group A—aneuploidies

Group A (164/902 patients, 18%) consisted of patients with aneuploidies. Referring clinicians were well acquainted with the phenotypic profile of patients with an aneuploidy, and the testing strategy available to confirm such a clinical diagnosis. Consequently, 61% of these patients had received a genetic confirmation test before their first visit to the genetics clinic, resulting in a negative time to diagnosis (mean −129 days and median  −29 days). Diagnostic yield was high in this group (97%), and the appropriate test was typically used (mean and median number of genetic tests performed was 1.1 and 1.0 respectively) – either QF-PCR (84%) or karyotype (16%). The mean and median costs to diagnose a patient with an aneuploidy were R1746,45 ($131,11) and R1707,21 ($128,17).

### Group B—clinically recognizable conditions

Patients in Group B (226/902; 25%) were those diagnosed by medical geneticists with conditions with well described clinical phenotypes. In 61% of these patients no genetic confirmation could be provided. In most cases, this was because there was no test available in the system to diagnose the condition in question. Next generation sequencing methodologies are not available routinely and variant- or disease specific assays are limited in terms of the number of conditions covered and knowledge of African pathogenic variants.

In some cases, geneticists were sufficiently confident in a patient’s clinical diagnosis that genetic confirmation was not considered essential to inform management. Two subgroups of patients were identified in Group B for whom geneticists were particularly confident in their clinical diagnosis. In the first, Group B1, (n = 29), diagnostic clinical investigations were available, for instance, confirmation of sickle cell anaemia through a haematological test, and in the second, Group B2, (n = 117), the conditions had a very clear distinct clinical phenotype, such as albinism. In these cases, clinicians considered the diagnoses firm enough to allow for appropriate counselling, future pregnancy risk assessment and condition specific management, without a confirmatory genetic test. The use of genetic tests in these two subgroups, and their associated costs, reflect this pattern of behaviour. The median and mean number of tests for the first group was 1 and 0,86 tests, with a median cost of R0,00 and a mean cost of R1079,25 ($81,02). The median and mean number of tests for the second group was 0 and 0,63 tests, with a median cost of R0,00 and a mean cost of R1119,52 ($84,04).

A third subgroup (Group B3) was identified (n = 80) who had phenotypes that were recognizable but not as clearly distinct as the first two groups, such as 22q11 syndrome or Noonan syndrome. These patients had suggestive diagnoses that could guide appropriate testing, but clinicians had lower confidence in clinical diagnoses, so relied more on genetic confirmation for diagnosis than in the first two subgroups of Group B. The median and mean number of tests in this group was 2 and 1,88 tests, and the median and mean costs were R3115,07 ($205,60) and R3226,42 ($212,94). The overall median and mean cost for Group B were R1467,95 ($110,21) and R1860,16 ($139,65). In this resource constrained context, patients with a greater need for testing are prioritised, and with fewer types of genetic investigations available, where there is a high degree of confidence in clinical diagnoses, genetic confirmation is not always pursued.

### Group C—rare, less recognizable conditions

Patients in Group C (512/902; 57%) presented with non-specific features that did not clearly point to a recognised condition. Of these patients, 90% presented with features of a DD – with developmental delay (55%), congenital anomalies (45%) and dysmorphic features (39%) most observed (Additional file 1: Fig. 2).

Only 8% (39/512) of patients in Group C received a diagnosis. 45 patients were considered lost to follow-up as they had not returned for a follow up visit or further testing by the end of the data capture period (~ 1–2 years after their first visit). These patients were removed from further analysis to prevent skewing of results.

**Fig. 4:**
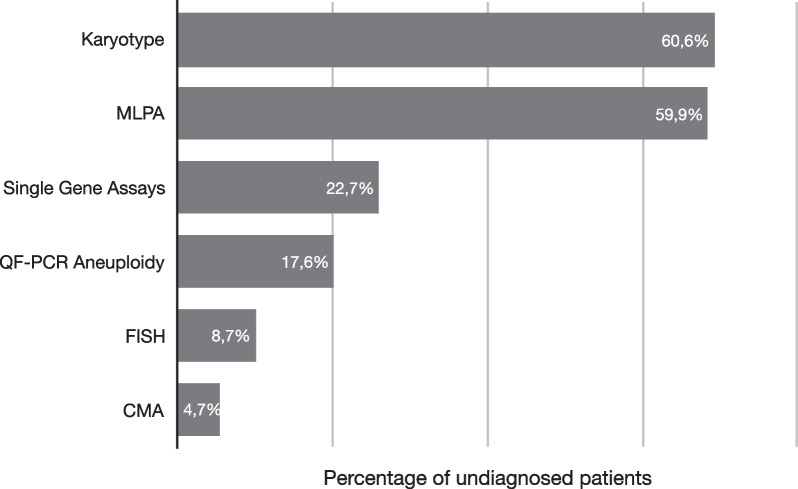
Genetic tests of undiagnosed patients in Group C to reach a diagnosis. The most common tests ordered for Group C patients were Karyotypes and MLPA for detection of sub-telomeric deletions/duplications and known microdeletion/duplication syndromes. Single gene assays include Fragment analysis and Sanger sequencing

The non-specific diagnostic tests used most commonly undertaken to provide diagnoses in this group included those able to detect chromosomal aberrations (for example karyotype, MLPA, for common micro-deletions and sub-telomeric deletions/duplications, and CMA) (Fig. 4)*.* Only 23% of these patients had single gene assays. QF-PCR aneuploidy was performed in 18% of patients, and FISH tests for specific micro-deletion and -duplication syndromes were undertaken in a minority (9%) of cases.

Many patients in Group C had numerous investigations in pursuit of a diagnosis: the median and mean number of tests per patient were 3 and 2,6 and more than half of patients (54%) had three or more tests (Additional file 1: Fig. 3).

### Cost of genetic investigations

**Fig. 5:**
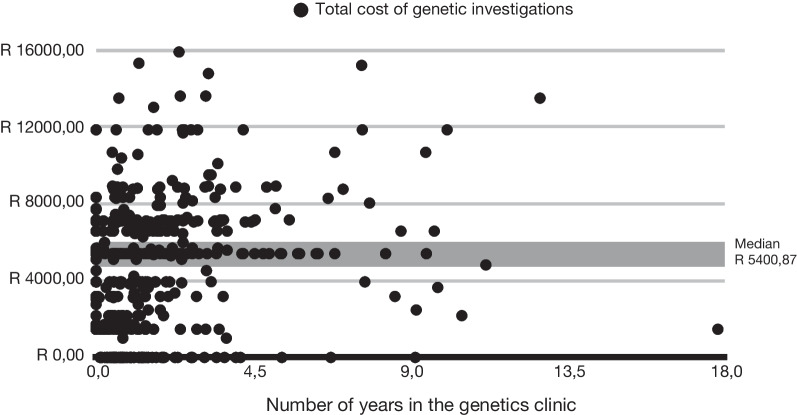
Total cost of genetics investigations for each Group C patient against the length of time the patient has been in the clinic**.** Many patients have multiple investigations with mounting costs over many years and yet remain undiagnosed. The median cost per patient was R5400,87 ($405,47)

Figure 5 shows the patients in Group C undergo multiple tests that return negative results, so remain in the clinic for several years awaiting a diagnosis. The median and mean cost of testing were R5400,87 ($405,47) and R4774,30 ($358,43) (range: R0,00—R15 895,58) per patient. These costs were significantly higher than those incurred by patients in Groups A and B. A comparison of costs between Groups A, B and C can be found in Additional file 2: Table 2. These costs do not include the cost of time with clinicians or the cost of other clinical investigations, such as radiological, haematological or metabolic testing, ordered as part of the diagnostic odyssey or for broad management principles. Consequently, the total cost per patient of seeking a diagnosis and on broad management is likely higher than these figures.

## Discussion

In this file audit of a cohort of patients presenting to a medical genetics clinic in South Africa over a one-year period, we found that the largest group of patients are those presenting with non-specific features of DD. These patients are not being effectively diagnosed by currently available testing methodologies and may benefit from the introduction of a test with a higher yield than those currently in use, such as exome sequencing. Exome sequencing can detect multiple types of variants across the genome and is now recommended by the ACMG as the first-line test for patients with DD [7].

The first group of patients identified (Group A) were those diagnosed with aneuploidies, with trisomy 21 being the most common diagnosis. This was expected as this is considered the most common chromosomal condition worldwide [16]. The diagnostic process for this group of patients in South Africa differs from the process in developed countries, where most diagnoses are made through prenatal screening, increasingly by non-invasive prenatal testing [17]. In this cohort, most patients were diagnosed postnatally by clinicians and then presented to the genetics clinic for genetic confirmation and/or counselling. This is most likely due to inconsistent prenatal screening for Down syndrome and other aneuploidies in antenatal care in South Africa [18]. Even though these patients were successfully diagnosed postnatally and do not require introduction of exome sequencing to improve diagnosis, this research highlights the fact that prenatal screening and diagnosis of aneuploidies is currently very limited in South Africa. Testing for aneuploidies should continue, where indicated, using QF-PCR.

The second group of patients (Group B) were those who could be diagnosed with confidence based on recognizable features, clinical diagnostic investigation or clinical scoring systems. This group was heterogenous, but most often diagnoses were sufficiently confident to inform management, regardless of whether genetic confirmation of the diagnosis was obtained. During the period of review, patients in this category did not always receive a genetic confirmation of the clinical diagnosis due to the limited type of genetic test available. It can be argued that exome sequencing could yield firm genetic diagnoses for many of these patients [19]. Even though these patients received clinical diagnoses, confirmed genetic diagnoses from exome sequencing within a shorter timeframe would be beneficial. Such diagnoses could enable more precise management due to the impact of genotype–phenotype correlation, and in time, possible gene or variant-specific targeted therapies. For families a quicker confirmed genetic diagnoses would allow more accurate prenatal risk assessment and could reduce psychosocial stress, by being certain of the genetic cause [20]. Additionally increasing the number of molecular diagnoses would assist in understanding the genetic epidemiology present in poorly characterized African populations.

The third and largest group of patients (Group C), were those who presented with non-specific features of DD. Only 8% of these patients received a diagnosis, on a par with the expected diagnostic yield of the traditional tests employed [21]. The investigation costs over time show how many of these patients remained on a diagnostic odyssey, having spent many years in genetics clinics with successive tests being done and yet remaining undiagnosed. Due to the limited number of tests available, in many cases the test ordered was not in fact the most appropriate test, and so had a high likelihood of returning a negative result. This results in mounting costs for these patients, probably underestimated in this report, as we did not capture the costs of other clinical investigations, time with clinicians or indirect costs to families. A recent study by Dragojlovic et al. suggests that the indirect costs of patients who remain undiagnosed should be considered when assessing the cost-effectiveness of new testing methodologies, such as exome sequencing. Their study shows that even if initial costs are higher, having a genetic diagnosis can result in lower indirect costs compared to patients who remain undiagnosed [22].

This study demonstrates that genetic diagnostic processes in use in South Africa are inadequate to diagnose patients with DD, and new methods must be introduced if the diagnostic rate is to improve. Routine use of CMA could be a first step to improving the diagnostic rate as this technique is known to have a diagnostic yield for DD of 15–20% [1]. However, CMA is not able to detect single nucleotide variants. Exome sequencing would therefore be the ideal tool to improve diagnostic yield in this context. The diagnostic yield of exome sequencing has been estimated at 30–53% for patients with NDD [4], and would be further enhanced if CNV analysis was also undertaken, as is increasingly possible [23].

Diagnostic exome sequencing is currently only available in South Africa to those who can pay privately for international diagnostic services. At around R15 000–35 000 ($1000–2 250); these costs are high and not a viable option for the majority of patients reliant on the State health system, considering the median monthly income in South Africa is R2800 ($210,21) per month. A small in-house evaluation projects the cost of implementing WES locally would cost of approximately ZAR13,000.00, and could potentially reach diagnostic rate as great as 70% [24]. For exome sequencing to be viable it will have to be performed locally and paid for by State services. The projected cost of a diagnostic exome sequence in South Africa is still relatively high due to the lack of established infrastructure, and high component and training costs [24]. Furthermore, interpreting the large amount of data produced by exome sequencing requires new skill sets and reliable pipelines to ensure optimal analysis. Additionally, there remains a high likelihood of finding variants of unknown/uncertain significance (VUS), which may not enable firm diagnoses to inform management [25]. The identification of VUS presents a specific challenge in an African context as limited baseline population data are available to reference when determining pathogenicity [26]. New validation and governance protocols will also be required to implement exome sequencing in clinical practice. Many of these challenges will only be overcome by capacity development through implementation and use over time, further underscoring the importance of timely implementation of exome sequencing as a first-line diagnostic option in LMICs [27].

A further challenge relates to how to make an economic case for the use of exome sequencing in this context in South Africa. There are currently no economic evaluations of the use of any form of exome or genome sequencing in an African context [28]. Such evidence is crucial given that countries across the continent have limited health budgets and many competing funding priorities, including treatments for infectious and chronic diseases that affect millions of people. The data presented in this paper, on testing costs and time to diagnosis, contribute to this economic evidence base. This is, however, only a first step towards generating the required evidence to support the implementation of exome sequencing in routine diagnostic services. Studies evaluating the cost-effectiveness of exome and genome sequencing in this setting are urgently required. These studies should go beyond a comparison of genetic testing costs, to consider the costs incurred by patients before and after testing, related to clinical care (both in primary care and secondary care). Importantly, such studies should also consider the full impact of a confirmed genetic diagnosis on the quality of life of patients and their families. The research by Masri and Hamamy [14] in Jordan, suggesting that exome sequencing may be cost effective in developing countries, and supports further investigation of the cost effectiveness of implementing diagnostic exome sequencing in this context.

## Conclusions

This retrospective audit of genetics clinic patients in South Africa provides insight into different groups of patients and how the current diagnostic process is serving them. In all groups there is a need to improve and upgrade testing. Although exome sequencing would not be the first-line option for a subset of patients with aneuploidies, and perhaps for some patients with common monogenic conditions, for most patients the implementation of exome sequencing would be the best way to attain broad-based genetic diagnoses. We conclude that exome sequencing has the potential to be a worthwhile investment in a low resource setting and will provide a much-needed leap forward in precision medicine and health improvement in these settings.

## Methods

### Case review selection

This proband-only cohort was selected from patients attending genetics clinics managed by the Division of Human Genetics, National Health Laboratory Services (NHLS) and The University of the Witwatersrand, in Johannesburg, South Africa. These clinics operate in the University of Witwatersrand academic hospitals that are part of the public health sector, which serves 80% of the South African population. Patients are referred to this medical genetics clinics from hospitals and non-genetics clinics in Johannesburg, and the southern Gauteng province, as well as from clinics in neighbouring provinces, where genetic services are not available. Genetic testing is offered via the NHLS, that serves the public health sector in a similar manner. Figure 1 summarises the structure of these clinics and the types of referrals received. In this study we distinguish between clinical- and genetic diagnosis, with genetic confirmation of a condition referring to cytogenetic or DNA-based testing methodologies.

Patients who attended medical genetics clinics between January to December 2017 were included in this study. Both new patients and patients returning for follow-up visits were included. All test results for these patients were included in assessing their diagnostic test, number of tests and cost calculations, whether or not they fell within the review period. Patients who attended fetal medicine and counselling clinics were excluded from this study as these are focused on counselling for high-risk pregnancies and screening or preventative genetic testing, not the diagnosis of an affected proband.

### Data capture and management

Human Genetics files were retrieved for each patient from the file archive at the Division of Human Genetics and data were extracted on proband demographics (including age, sex and ethnicity), clinical phenotype and assessments by clinic geneticists, and information from other specialists relevant to genetic assessment and management. Genetic test results were accessed via the online laboratory results system NHLS LabTrak. Data were captured and managed using REDCap, hosted at the University of Witwatersrand [29, 30].

### Diagnostic costing

The cost of genetic testing was calculated by combining information on resource use (the diagnostic tests captured for each proband) and test prices extracted from the 2017 NHLS State Price list. This in-house list presents prices agreed between the South African Department of Health and the NHLS. The prices applied in this study are listed in Additional file 2: Table 3, with ZAR to USD conversion calculated according to average exchange rate for 2017 (R13,32 = $1). One example of a diagnosis made from a test performed as part of research project was included in the dataset, but as this was not performed and charged by NHLS it was not included in the costing analysis. Test cost data were summarised using means, medians and ranges.

### Data analysis

Data cleaning, analysis and visualization were performed in Stata13 (StataCorp. 2013. Stata Statistical Software: Release 13. College Station, TX: StataCorp LP.) and Apple Numbers (version 6.2.1. Apple Inc. California).

## Supplementary Information

**Additional file 1.** This file contains all supplementary figures.

**Additional file 2.** This file contains all supplementary tables.

## Data Availability

The datasets generated and/or analysed during the current study are not publicly available due to the fact that patients file records may only be analysed as anonymous records by department researchers and published as aggregate results unless specific informed consent is gained from the patient, parents or guardians for publication or other use of their data. De-identified data are available upon request from the corresponding author.

## References

[1] Miller DT, Adam MP, Aradhya S, Biesecker LG, Brothman AR, Carter NP (2010). Consensus statement: chromosomal microarray is a first-tier clinical diagnostic test for individuals with developmental disabilities or congenital anomalies. Am J Hum Genet.

[2] Mithyantha R, Kneen R, McCann E, Gladstone M (2017). Current evidence-based recommendations on investigating children with global developmental delay. Arch Dis Child.

[3] Carmichael N, Tsipis J, Windmueller G, Mandel L, Estrella E (2015). "Is it going to hurt?": the impact of the diagnostic odyssey on children and their families. J Genet Couns.

[4] Srivastava S, Love-Nichols JA, Dies KA, Ledbetter DH, Martin CL, Chung WK (2019). Meta-analysis and multidisciplinary consensus statement: exome sequencing is a first-tier clinical diagnostic test for individuals with neurodevelopmental disorders. Genet Med.

[5] Li C, Vandersluis S, Holubowich C, Ungar WJ, Goh ES, Boycott KM (2021). Cost-effectiveness of genome-wide sequencing for unexplained developmental disabilities and multiple congenital anomalies. Genet Med.

[6] Bourchany A, Thauvin-Robinet C, Lehalle D, Bruel AL, Masurel-Paulet A, Jean N (2017). Reducing diagnostic turnaround times of exome sequencing for families requiring timely diagnoses. Eur J Med Genet.

[7] Manickam K, McClain MR, Demmer LA, Biswas S, Kearney HM, Malinowski J (2021). Exome and genome sequencing for pediatric patients with congenital anomalies or intellectual disability: an evidence-based clinical guideline of the American College of Medical Genetics and Genomics (ACMG). Genet Med.

[8] Tan TY, Dillon OJ, Stark Z, Schofield D, Alam K, Shrestha R (2017). Diagnostic impact and cost-effectiveness of whole-exome sequencing for ambulant children with suspected monogenic conditions. JAMA Pediatr.

[9] Maulik PK, Mascarenhas MN, Mathers CD, Dua T, Saxena S (2011). Prevalence of intellectual disability: a meta-analysis of population-based studies. Res Dev Disabil.

[10] Global Research on Developmental Disabilities C. Developmental disabilities among children younger than 5 years in 195 countries and territories, 1990–2016: a systematic analysis for the Global Burden of Disease Study 2016. Lancet Glob Health. 2018;6(10):e1100-e21. 10.1016/S2214-109X(18)30309-7 PMC6139259 30172774

[11] Kromberg JG, Sizer EB, Christianson AL (2013). Genetic services and testing in South Africa. J Commun Genet.

[12] Thong MK, See-Toh Y, Hassan J, Ali J (2018). Medical genetics in developing countries in the Asia-Pacific region: challenges and opportunities. Genet Med.

[13] Tsang MHY, Chiu ATG, Kwong BMH, Liang R, Yu MHC, Yeung KS (2020). Diagnostic value of whole-exome sequencing in Chinese pediatric-onset neuromuscular patients. Mol Genet Genomic Med.

[14] Masri A, Hamamy H. Cost effectiveness of whole exome sequencing for children with developmental delay in a developing country: a study from Jordan. J Paediat Neurol. 2021:s-0040–1722265.

[15] Statistics South Africa General Household Survey. 2021. https://www.statssa.gov.za/?p=15482.

[16] Bull MJ (2020). Down Syndrome. N Engl J Med.

[17] Rudolf G, Tul N, Verdenik I, Volk M, Brezigar A, Kokalj Vokac N (2017). Impact of prenatal screening on the prevalence of Down syndrome in Slovenia. PLoS ONE.

[18] Urban MF, Stewart C, Ruppelt T, Geerts L (2011). Effectiveness of prenatal screening for Down syndrome on the basis of maternal age in Cape Town. S Afr Med J.

[19] Dillon OJ, Lunke S, Stark Z, Yeung A, Thorne N, Melbourne Genomics Health A, et al. Exome sequencing has higher diagnostic yield compared to simulated disease-specific panels in children with suspected monogenic disorders. Eur J Hum Genet. 2018;26(5):644–51. 10.1038/s41431-018-0099-1 PMC5945679 29453417

[20] Makela NL, Birch PH, Friedman JM, Marra CA (2009). Parental perceived value of a diagnosis for intellectual disability (ID): a qualitative comparison of families with and without a diagnosis for their child's ID. Am J Med Genet A.

[21] Challman TD, Barbaresi WJ, Katusic SK, Weaver A (2003). The yield of the medical evaluation of children with pervasive developmental disorders. J Autism Dev Disord.

[22] Dragojlovic N, van Karnebeek CDM, Ghani A, Genereaux D, Kim E, Birch P (2020). The cost trajectory of the diagnostic care pathway for children with suspected genetic disorders. Genet Med.

[23] Coe BP, Witherspoon K, Rosenfeld JA, van Bon BW, Vulto-van Silfhout AT, Bosco P (2014). Refining analyses of copy number variation identifies specific genes associated with developmental delay. Nat Genet.

[24] Flynn KA. Evaluating whole exome sequencing on the Ion Torrent S5™ as a potential diagnostic tool for developmental disorders [Unpublished Degree type thesis or dissertation]. Johannesburg: University of Witwatersrand; 2020. Available from: https://wiredspace.wits.ac.za/handle/10539/45

[25] Krause A (2019). New genetic testing technologies: advantages and limitations. S Afr Med J.

[26] Bope CD, Chimusa ER, Nembaware V, Mazandu GK, de Vries J, Wonkam A (2019). Dissecting in silico mutation prediction of variants in African genomes: challenges and perspectives. Front Genet.

[27] Kamp M, Krause A, Ramsay M (2021). Has translational genomics come of age in Africa?. Hum Mol Genet.

[28] Schwarze K, Buchanan J, Taylor JC, Wordsworth S (2018). Are whole-exome and whole-genome sequencing approaches cost-effective? A systematic review of the literature. Genet Med.

[29] Harris PA, Taylor R, Thielke R, Payne J, Gonzalez N, Conde JG (2009). Research electronic data capture (REDCap)–a metadata-driven methodology and workflow process for providing translational research informatics support. J Biomed Inform.

[30] Harris PA, Taylor R, Minor BL, Elliott V, Fernandez M, O'Neal L (2019). The REDCap consortium: building an international community of software platform partners. J Biomed Inform.

