# Supplementary material for: Retrospective file review shows limited genetic services fail most patients – an argument for the implementation of exome sequencing as a first-tier test in resource-constrained settings
Source: Orphanet J Rare Dis. 2023 Apr 12;18:81. doi: 10.1186/s13023-023-02642-4 (PMC10091645; doi:10.1186/s13023-023-02642-4)
Supplement: Supplementary file 1 — Additional file 1. This file contains all supplementary figures. [file 13023_2023_2642_MOESM1_ESM.pdf]

Supplementary Figures

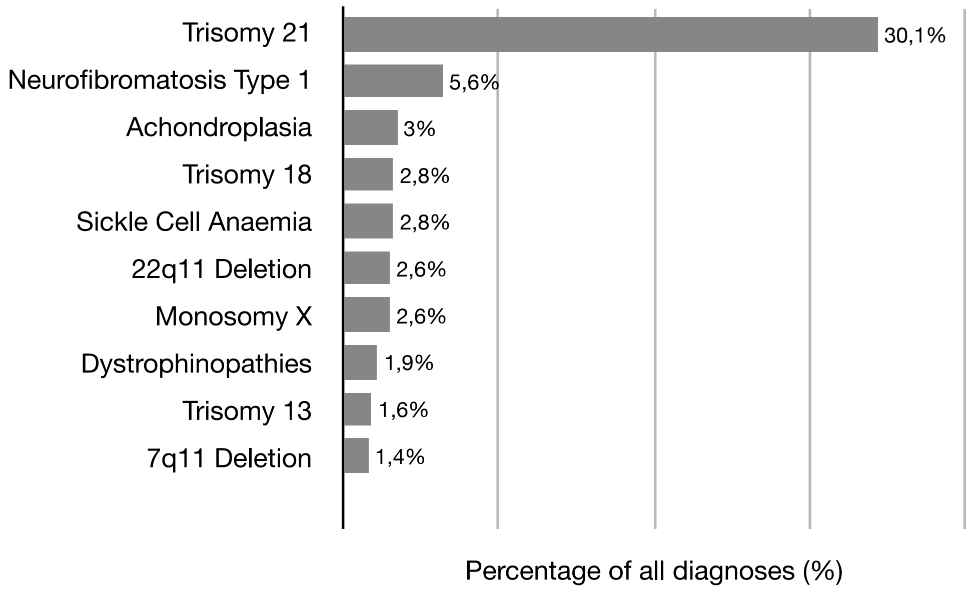

Supplementary Figure 1: Top 10 genetic diagnoses made in the cohort. These top ten diagnoses account for 54% of all diagnoses, with Trisomy 21 being the most common diagnosis made.

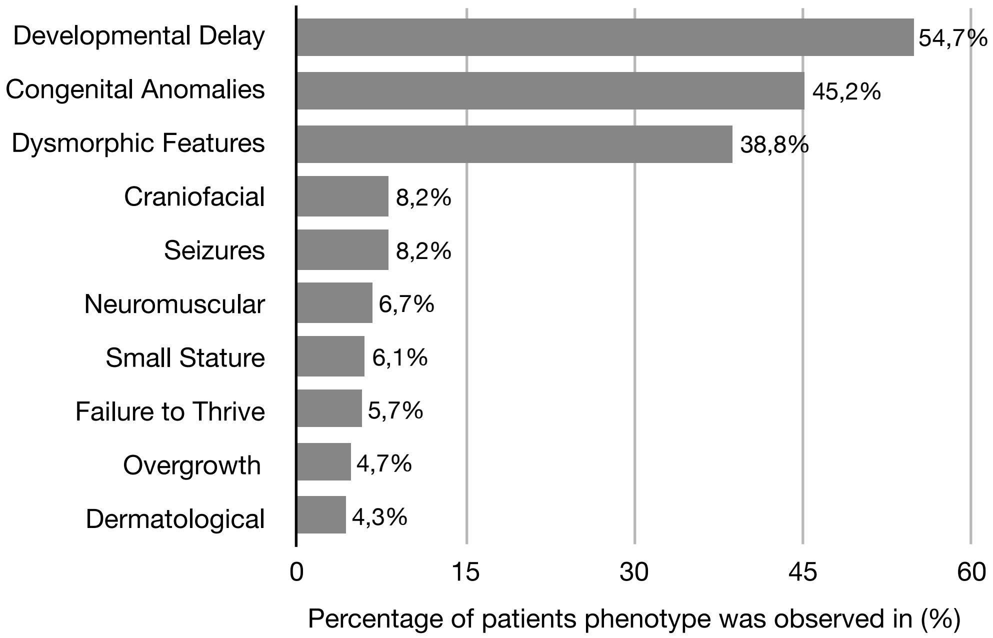

Supplementary Figure 2: Top ten phenotypes observed in patients in Group C The most common phenotypes by far were developmental delay, congenital anomalies and dysmorphic features.

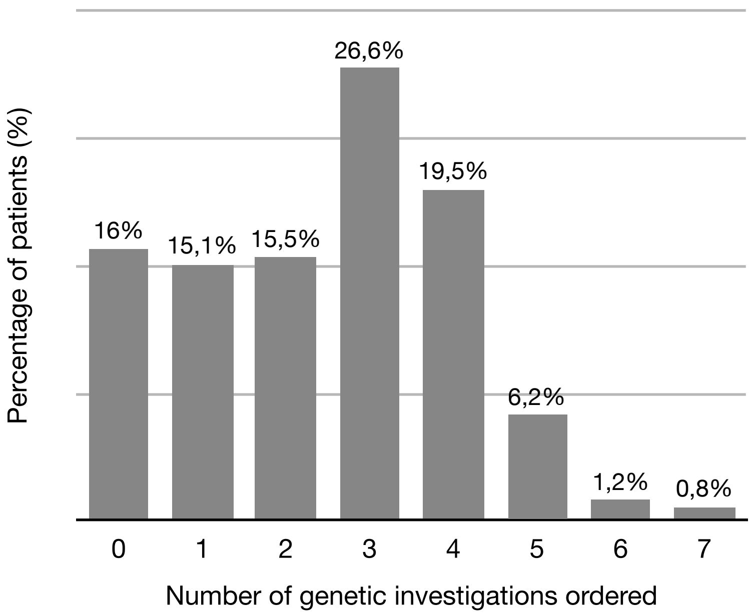

Supplementary Figure 3: Number of genetic investigations undergone by undiagnosed patients in Group C. The greatest percentage (26,6%) of patients had 3 tests ordered and 54.3% of patients had 3 or more tests.
